# Reward prediction error in the ERP following unconditioned aversive stimuli

**DOI:** 10.1038/s41598-021-99408-4

**Published:** 2021-10-07

**Authors:** Harry J. Stewardson, Thomas D. Sambrook

**Affiliations:** School of Psychology, University of East Anglia, Norwich Business Park, NR4 7TJ UK

**Keywords:** Neuroscience, Psychology

## Abstract

Reinforcement learning in humans and other animals is driven by reward prediction errors: deviations between the amount of reward or punishment initially expected and that which is obtained. Temporal difference methods of reinforcement learning generate this reward prediction error at the earliest time at which a revision in reward or punishment likelihood is signalled, for example by a conditioned stimulus. Midbrain dopamine neurons, believed to compute reward prediction errors, generate this signal in response to both conditioned and unconditioned stimuli, as predicted by temporal difference learning. Electroencephalographic recordings of human participants have suggested that a component named the feedback-related negativity (FRN) is generated when this signal is carried to the cortex. If this is so, the FRN should be expected to respond equivalently to conditioned and unconditioned stimuli. However, very few studies have attempted to measure the FRN’s response to unconditioned stimuli. The present study attempted to elicit the FRN in response to a primary aversive stimulus (electric shock) using a design that varied reward prediction error while holding physical intensity constant. The FRN was strongly elicited, but earlier and more transiently than typically seen, suggesting that it may incorporate other processes than the midbrain dopamine system.

## Introduction

A challenge facing neuroscientific studies of reward prediction error (RPE) is linking research at the microscopic and mesoscopic scales. Single cell recordings have demonstrated RPE encoding in midbrain dopamine (DA) neurons, occurring with a latency of 50–110 ms and peaking at 200 ms^1–3^. Meanwhile, the event related potential technique has demonstrated a frontocentral component, variously known as the feedback related negativity (FRN) or reward positivity, claimed to encode an RPE, and occurring with a latency of 150 ms, peaking at around 280 ms^4,5^. The FRN is localised most frequently to the cingulate cortex, though sometimes to the striatum^6^, both targets of midbrain DA neurons. A pertinent question is whether the FRN reflects the transmission of a midbrain RPE signal to corticolimbic structures, as one prominent theory claims^7^.

Midbrain DA cells respond to both appetitive and aversive stimuli, coding positive RPEs (e.g. delivered juice, omitted air puff) with increased phasic firing, and negative RPEs (e.g. omitted juice, delivered air puff) with phasic dips^3,8^. This implies that diverse motivational events may be encoded on a single scale of RPE utility. In the present paper we accordingly use the term RPE to cover cases of negative (e.g. delivery of electric shock) as well as positive utility. A further notable characteristic of midbrain DA cells is that they respond both to unconditioned stimuli (e.g. air puff, juice), and conditioned stimuli that indicate imminent delivery of these primary reinforcers^9^. This accords with a temporal difference learning model in which RPE signals that are initially incurred at receipt of a primary reinforcer propagate back in time to the presentation of a conditioned stimulus predicting that reinforcer’s delivery^10,11^. The similarity of neural response to conditioned and unconditioned stimuli suggests that properties of the actual stimulus are not encoded by these cells, merely its motivational *value*, and so a process of simple model-free learning reinforcement learning is at work^11,12^.

If the FRN arises from the transmission of this signal to the basal ganglia and cortex, then the same equivalent response to unconditioned or conditioned stimuli should be seen in this component. However, very little is known about the FRN’s response to unconditioned stimuli. While some FRN studies have used primary reinforcers such as food or shock rather than the ubiquitous secondary reinforcer of money, the FRN is near universally measured not in response to these reinforcers but to a cue denoting their subsequent delivery. This cue may be a fully abstract “truth cue” (e.g. a geometric shape), may carry a conventional valence denotation (e.g. a cross or tick) or, less frequently, may visually reference the reinforcer (e.g. a picture of a snack). Over the course of an experiment, this cue should be expected to acquire the status of a conditioned stimulus, and so should be expected to engage the midbrain DA cells. Initially, however, the correspondence between the cue and the reinforcer it denotes must be consciously apprehended by participants as a result of instruction and held in working memory. While it is possible that such a mapping is routed via the midbrain DA system from the outset in order to create an RPE, this need not be so, and the FRN in this case might not reflect an RPE in the strict model-free sense, but rather a more generic performance error. That the FRN might reflect quantities other than RPE is supported on a number of grounds. The component can be elicited, albeit in attenuated form, when feedback denotes a correct response, but one to which no reward is attached^13,14^. It is shown by participants observing rewards or punishments incurred by others^15,16^. There is also evidence that it incorporates counterfactual knowledge about unchosen options, something difficult to reconcile with model-free reinforcement learning^17^. Thus, the FRN, as traditionally operationalised, may represent the aggregation of overlapping, same-polarity deflections associated with functionally and anatomically distinct processes broadly relating to error and success. Alternatively, it may reflect a single unitary response that integrates such inputs, including both RPE and performance monitoring aspects. In either case however, if at least some portion of the FRN is derived from activity of the midbrain DA neurons, as has been claimed, it should be expected to be sensitive to the direct presentation of unconditioned stimuli in the manner shown by these cells.

Measuring the event related potential to a primary reinforcer, rather than to a cue, presents methodological challenges. The use of cues is pragmatic insofar as it makes it easy to study RPE while controlling out differences in the sensory properties of unconditioned stimuli. This includes both their modality (electric shocks might produce different event related potentials from money) and intensity (large shocks might produce larger potentials than their small equivalents even when the RPE they carry is equivalent). Locking the waveform to arbitrary truth cues, counterbalanced across the reinforcers they denote, deals with the possibility of sensory confounds which can otherwise never be ruled out. Additionally, it may attenuate attentional, orienting responses that are specific to the onset of primary reinforcers, and which have nothing to do with RPEs or learning. This should produce better signal to noise ratio and, indeed, the FRN as locked to cues is a robustly elicited component.

Measuring RPE in response to an unconditioned stimulus faces two principal challenges. One of these is to present the stimulus at a sufficiently well specified time in order to align epochs and so resolve components in the averaged waveform. Food or drink ingestion is difficult to time-lock in this way, as are naturally occurring social rewards such as smiling or laughter. Studies that have attempted to use affective images to generate the FRN have fared poorly^18,19^, perhaps due to weak operationalisation of those images via participant ratings. In the aversive domain, which is less often used in studies of human reinforcement learning, two temporally precise reinforcers are available however, electric shocks and bursts of white noise.

A second problem with the use of unconditioned stimuli, alluded to earlier, is that RPE is typically confounded with *physical salience*, that is the intensity of the stimulus^20^. As the physical salience of a motivationally relevant stimulus increases (e.g. sweetness of juice, strength of shock), its utility, in terms of RPE, changes too, with the direction depending on whether the stimulus is appetitive or aversive, and it therefore becomes unclear whether RPE or physical salience underlies the observed potential. The FRN is typically operationalised as a negative going bad–good difference wave, indicating a positive relationship between RPE utility and voltage. In the case of an aversive stimulus such as shock, lower than expected shocks should produce a relative positivity of voltage compared to that produced by greater than expected shocks, producing a negative going high shock–low shock difference wave. However, when the FRN is locked to the actual shock, rather than a cue, such an effect could equally as well be cited as evidence for a purely sensory component responding to physical salience, in this case via voltage negativity for increasing salience. Since RPEs are rarely the object of study in experiments using electric shock, this confound is typically not addressed. However, one study removed the confound by delivering shocks of equal intensity in which one shock was higher than expected (negative RPE) and the other lower (positive RPE). In this study, lower than expected electric shocks produced a waveform showing a relative positivity from 60 ms onwards, although a significant effect was only reported in the context of a late P2 component, at approximately 350 ms, allowed to vary over site and latency across the fourteen participants^21,22^. Using a similar design, though with laser evoked potentials, and only six participants, another study found encoding of physical salience, but inspection of the tabulated data suggests no RPE effect^23^. Importantly, both studies cited above delivered stimuli to participants who were passive in the process, but the FRN is known to be weak or absent in such tasks^6^. The aim of the present study was to assess the evidence that the FRN is elicited in response to RPEs carried by an unconditioned stimulus in an active learning task.

Satisfying such a claim would require a demonstration of neural activity consistent with the FRN’s polarity, topography and latency. For polarity, positive RPEs would need to show a positive voltage relative to negative RPEs, thus producing the negative going bad–good difference wave by which the FRN is conventionally operationalised. Topographically, the component should be frontocentral. Temporally, it should have a similar latency to the FRN as typically reported. This latency is dependent on the terms by which the FRN is defined, however. While, as noted above, the standard operationalisation is a bad–good valence contrast, because of interest in the FRN’s role in coding a quantitative RPE rather than merely a dichotomous comparison of RPE sign, the FRN is also sometimes operationalised in terms of the difference of this difference wave for small and large RPEs^6,7,24–26^. A meta-analysis of the FRN showed that while both operationalisations peaked at around 280 ms, they showed nested intervals, with the FRN lying between 150 and 400 ms, and the difference of difference waves lying between 240 and 340 ms^5^. Since the present design did not manipulate RPE size, the wider interval was selected as the interval of interest.

In addition to investigating the effect of primary reinforcers on the FRN, the present study’s use of electric shock supports an investigation of affective aspects of RPE encoding. RPEs consist in a simple differencing of expected and received value. The encoding of value implies a motivational and therefore affective element, however the functional links between RPE generation and wider affect are not fully understood. Early accounts of the FRN varied in the degree to which they stressed its affective^17^ versus computational qualities^7^. While self-reported affect does not correlate well with FRN amplitudes^27–29^, physiological measures provide a plausible alternative, provided reinforcers are sufficiently strong to produce a measurable response, as might be expected for shock. The present study used the startle response to unexpected bursts of white noise to index the affective state of participants waiting in anticipation of shock. The startle response was chosen partly because it is used across multiple mammal species, but also because it has been shown in humans to serve as a *bivalent* measure of affect, potentiated when participants face the prospect of an aversive outcome such as shock, and attenuated when they are shortly to receive an appetitive outcome such as sweet food^30^. This bivalent nature makes it ideal for capturing variance in the FRN which may be modulated by either positive or negative RPE.

## Methods

### Participants

Forty-nine students (20 males) of the University of East Anglia participated for course credit. All participants were under 29 years, had no history of neurological damage or other significant health problems, and were not on medication at the time of the experiment. Four participants failed to complete the experiment because they found the procedure too painful, and one because of equipment failure. The study was approved by the ethics committee of the School of Psychology at the University of East Anglia and methods were performed in accordance with the Declaration of Helsinki. The experiment was undertaken with the understanding and written consent of each participant, with participants receiving course credit for their participation.

### Shock apparatus

Shocks were delivered by electrocutaneous stimulation using a linear isolated stimulator (Model: STMISOLA; 45 BIOPAC Systems, Inc.) charged by a stabilized current, and electrodes (Model: EL350; BIOPAC Systems, Inc.) attached to the wrist of the participant’s non-dominant hand. Shocks were controlled from E-Prime code running on the presentation computer, communicating with the stimulator by means of a Measurement Computing Corporation (MCC) data acquisition device (USB-1208HS-4AO). Shock was carried by a 25 Hz sine wave lasting 160 ms and was delivered at three voltages on the basis of the calibration procedure described below.

### Shock calibration

In order to calibrate the aversiveness of shock on an individual basis, participants were administered a range of shock intensities and rated each on a ten-point visual analogue scale (see Supplementary Fig. S1). Shocks began at 30 V and were incremented in three-volt steps until the participant rated the pain at “8”, or 70 V was reached. The shock was decremented two volts, and then in steps of three volts until a rating of “3” was reported. Shock was then incremented by a single volt, and then in steps of three volts, until the participant rated the pain at “8”, or 70 V was reached. This procedure resulted in ratings at one-volt steps. Small, medium and large shocks for the subsequent experimental session were calculated by taking the mean voltage given a rating of “3” (“very slightly unpleasant”), “5” (“moderately unpleasant”) and “7” (“highly unpleasant but still tolerable for the forthcoming experiment”).

### Design

Although three shock sizes were used, these were implemented in a 2 × 2 trial stakes × RPE design. In low stakes trials, participants could receive either the small or medium shock, while in high stakes trials they could receive either the medium or large shock. Once participants had been so cued of the two permissible shocks, the arrival of one therefore represented a positive RPE and the arrival of the other a negative RPE. Cells in the design are hereafter denoted as low stakes positive (LP), low stakes negative (LN), high stakes positive (HP) and high stakes negative (HN). In terms of physical salience (a variable that was not present in the design, and which we wished to control out), LP shocks were small, LN and HP shocks were medium, and HN shocks were large. The critical contrast for the experiment was between LN and HP since this varies RPE while holding physical salience constant.

A power analysis was performed based on a meta-analysis of the cue-locked FRN^5^ in which the FRN was defined by the mean amplitude of the good–bad difference wave in the interval 228–334 ms. This meta-analysis used original subject-level data in 27 studies that were supplied by authors. The standardised effect sizes ranged from 0.19 to 2.03, with all FRNs correctly signed. The average standardised effect size was 1.00 (0.93 when this average was weighted by study sample size) and the average simple effect size in microvolts was − 2.16. Effect sizes of *treatment effects* on the FRN were of course much smaller but the present study was concerned merely with demonstrating the presence of an FRN.

Given an effect size of 1.00, an alpha of 0.05 and significance assessed with a one-sample *t* test, a manual calculation of power^31^ reveals that a sample size of 8 is required to achieve 0.8 power and 18 to achieve 0.99 power (10 and 21 participants if effect size is set to 0.93). However, the estimated effect size of 1.00 was based on studies that used a larger number of trials than were used in the present study. For ethical reasons the number of large shocks was limited to fifty per participant, resulting in two hundred trials per participant and one hundred trials in the critical comparison of LN and HP. The effect that varying trials has on effect size has been studied using the lateralised readiness potential^32^, a component with a comparable but somewhat lower simple effect size than the FRN. This study showed that when N > 15, increasing trials beyond 30 had negligible effects on internal consistency of the component’s amplitude. This finding tallies with a study specifically of the FRN which showed that with a sample size of 25, 20 trials were sufficient to measure FRN amplitude^33^. Because the effect size of the FRN in response to unconditioned stimuli was unknown, a sample size of 45 was selected to provide a comfortable margin of power.

### Procedure

**Figure 1:**
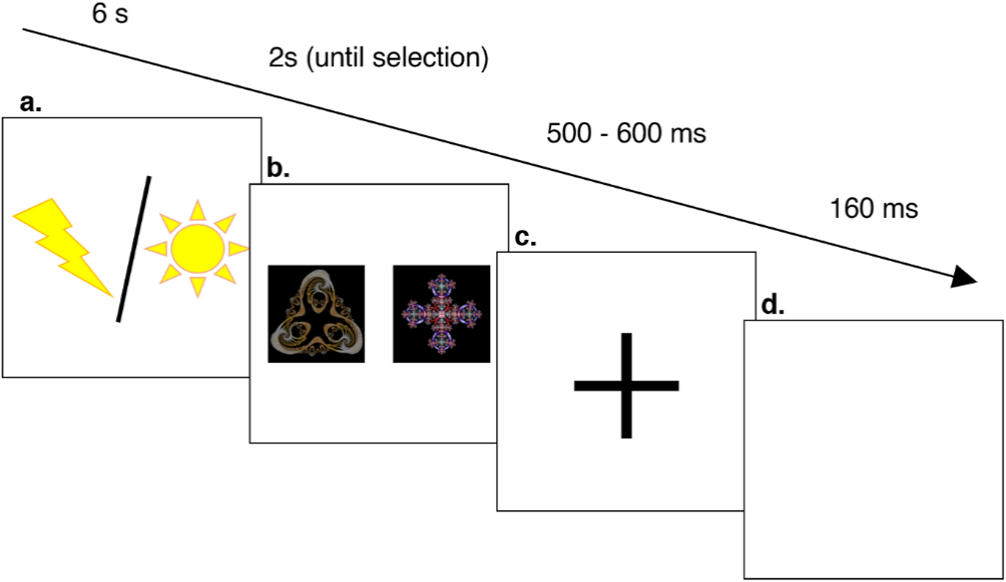
(**a**) Icon indicates if trial is high or low stakes, with noise administered on 20% trials at a random point between 2 and 5 s. (**b**) Participant selects from a pair of fractals. (**c**) Cross indicates shock is coming. (**d**) Shock is delivered.

Participants were informed that they would take part in a learning task in which they had to choose between two fractals and attempt to minimise their exposure to painful electric shocks that followed their choice. They were told to monitor the shock received and use that to guide their subsequent choices. The meaning of the stakes cues was explained to them and therefore which two levels of shock were possible on a given trial. They were told they would experience bursts of white noise, which they should ignore since these were unrelated to the task. The sequence of events on a trial is shown in Fig. 1. Participants were first presented with a stakes cue indicating whether the trial would be high or low stakes. This cue was visible for six seconds and on 20% of occasions participants were exposed to white noise during its presentation. On these occasions a 105 dB burst of white noise was delivered via headphones for one second during a randomly determined interval from two to five seconds. This was followed by the presentation of a pair of fractals (unique to each level of stakes, and each block of the experiment), which remained on screen for 2 s or until participants selected one of the fractals with a keypress. This keypress was the sole choice made by participants in a trial. This was followed by an image of a cross lasting 500–600 ms and then the delivery of a shock. Unbeknownst to participants, shock outcomes were predetermined such that half of the high stakes trials resulted in a large shock and half in a medium shock, presented in a pseudorandom order. Half of the low stakes trials resulted in a small shock and half in a medium shock. Keypresses therefore had no relationship with the size of the shock that was administered. Despite the task being unsolvable, participants were instructed to try and work out which key would minimise shock.

Participants undertook five blocks of forty trials each, with each block containing twenty high, and twenty low stakes trials, presented in a random order. The symbols denoting whether a trial was high or low stakes were held constant for a given participant but counterbalanced across participants. At the conclusion of each block, participants were asked which key they considered to have been most favourable. Since one key would generally be associated with lower shock by chance association, this served as an index of participants’ learning effort in the absence of a genuinely learnable task.

### EEG recording

EEG data were collected from 61 Ag/AgCl active electrodes (actiCAP, Brain Products, Gilching, Germany) mounted on an elastic cap and arranged in a standard International 10–20 montage referenced to the left mastoid. Vertical eye movement was monitored by a right suborbital electrode, and horizontal eye movement was monitored using an electrode on the right external canthus. Electrode impedances were kept below 20 kΩ. EEGs were amplified using a BrainAmp amplifier (Brain Products), continuously sampled at 500 Hz.

### EEG preprocessing

Data were filtered with notch filters at 60 Hz and 50 Hz, followed by a 0.1 Hz high pass filter and 30 Hz low pass filter. Segments were time-locked to 300 ms before the onset of shock to 700 ms afterwards and were baseline-corrected using the interval – 200 to 0 ms. Eye movement artefacts were removed using the semi-automatic ICA-based ocular artefact rejection function within BrainVision Analyzer software. Other non-specific artefacts in the interval – 200 to 700 ms were removed using a criterion of 50 μV change per ms, a voltage value exceeding 100 μV relative to baseline, or activity across the epoch below 12 μV. Segments were re-referenced to the average of left and right mastoid. Electrodes which malfunctioned in the course of an experiment (an average of 0.56 per participant) were substituted using topographic interpolation^34^. Data were further down-sampled to 100 Hz for the cluster-randomisation procedure described below. Mean trials per condition were as follows: LP, 48.59 (SD = 1.99), LN, 48.02 (SD = 3.22), HP, 47.73 (SD = 3.89), HN, 47.23 (SD = 4.69).

### Analysis methods

Two analysis strategies were employed to reveal RPE encoding. The first strategy was to assess the evidence for a significant LN–HP difference wave. This represents a comparison of negative RPEs and positive RPEs, thus conforming to a conventional analysis of the FRN, and controls for physical salience. The full waveform was analysed for evidence of such a difference wave, and cluster randomisation using the method of Maris and Oostenveld^35^ was used to compensate for multiple comparisons (see Fieldtrip^36^ version 20200607). In this process, one sample *t* tests, performed over participants, were used to establish significance of the difference wave at each sample (i.e. electrode × timepoint combination), and clusters of spatially and temporally adjacent significant samples were created (minimum 25). Each cluster was assigned a Monte Carlo *p* value based on positioning its sum of *t* values in a null distribution of clusters created from 400,000 permutations of the data under Monte Carlo simulation. The Monte Carlo *p* value returned by this procedure was Bonferroni-corrected by the number of clusters found at the initial stage. All *t* tests reported in this paper underwent this correction for multiple comparisons. On an exploratory basis, the same procedure was used to identify the timing and location of activity encoding physical salience. The contrast used for this purpose was high versus low stakes trials since this varies the aggregate level of physical salience while ensuring equal frequency of positive and negative prediction errors. Finally, it should be noted that no comparison was made between FRNs to high and low stakes (i.e. LN–LP vs. HN–HP) since these difference waves confound prediction error with stimulus intensity, the effects of which cannot be assumed to increase linearly over the range used in the experiment.

The second strategy employed a more fine-grained analysis of the evidence for RPE encoding in the context of what were expected to be strong effects of physical salience and the possibility that RPE encoding would behave differently under high and low stakes conditions. To separate out effects of RPE and physical salience encoding, we specified, for each of the six pairwise contrasts available from the 2 × 2 design, the expected direction of effect that would be produced by five possible encoders. These encoders were: RPE coded by either negative or positive voltage shifts (with positive shifts corresponding to the conventional FRN), physical salience encoded by either negative or positive voltage shifts, and a null encoder in which neither property was encoded. Evidence for these encoders’ activity at a given point in the waveform thus consisted in the degree to which the observed contrasts met the predictions. These predictions are given in Table 1. In a number of contrasts the predicted effect is that of no effect and since this is as diagnostically meaningful as an effect, a Bayesian approach was taken to place testing of the two possibilities on an equal footing. One-sample Bayesian *t* tests were used to test each contrast. These were run at each sample using the ttestBF function of the BayesFactor package^37^ in R, and the resulting Bayes’ factors were expressed as posterior probabilities in support of each of the predictions shown in the cells of Table 1. Evidence for each encoder was calculated by taking the product of the posterior probabilities of the six predicted effects specified in that encoder’s column. This evidence was normalised across encoders by dividing each encoder’s compound probability by the sum of the compound probabilities for all five encoders. The resulting probabilities, which summed to one, thus represented the *relative* evidence for each encoder compared to the others, and in particular the null encoder. Clusters of activity associated with each encoder were determined by thresholding these normalised compound probabilities at 0.975, making their significance comparable with the standard *t* tests used elsewhere in the paper.

**Table 1:** Rows designate the six pairwise contrasts available from the design.

Condition contrast	Salience contrast	RPE contrast	Encoding of				
			RPE		Physical salience		Nothing
			Voltage positivity	Voltage negativity	Voltage positivity	Voltage positivity	
HN/HP	High/med	Bad/good	HN < HP	HN > HP	HN > HP	HN > HP	HN = HP
HN/LN	High/med	Bad/bad	HN = LN	HN = LN	HN > LN	HN > LN	HN = LN
HN/LP	High/low	Bad/good	HN < LP	HN > LP	HN > LP	HN > LP	HN = LP
HP/LN	Med/med	Good/bad	**HP > LN**	HP < LN	HP = LN	HP = LN	HP = LN
HP/LP	Med/low	Good/good	HP = LP	HP = LP	HP > LP	HP > LP	HP = LP
LN/LP	Med/low	Bad/good	LN < LP	LN > LP	LN > LP	LN > LP	LN = LP

Columns 4–8 show the predicted effects that five different encoders will show in each contrast. The quantity compared in these contrasts is positivity of voltage. The emboldened cell identifies the single critical contrast used for the first analysis strategy.

For startle response analysis, EEG data were filtered with a 28 Hz high pass filter and notch filters at 60 Hz and 50 Hz, and rectified to isolate high frequency electromyographic effects^38^. Startle response was measured at the right suborbital electrode. Activity at this site was averaged in a rolling 50 ms window^30^ and baseline corrected from − 50 to 0 ms. Segments were removed as artefactual if activity exceeded ± 5 μV in the baseline (4.89% of trials) and removed as non-startle if no activity exceeding ± 5 μV was found in the interval 0–200 ms (49.45% of remaining trials). Remaining startle trials (mean per participant 17.73, range 2–36) were averaged for each participant to provide participant average startle response waveforms. Because two participants showed no startle responses in one of the two stakes conditions, they were excluded from the analysis of the effects of stakes on startle. Because of the great variation in the number of startle trials per participant, *t* tests and correlations seeking to establish group level effects were weighted by the number of blink trials provided by each participant. Significance was measured at a conventional α = 0.05 (two tailed) and no outliers were found for any of the analyses when assessed against a criterion of z > 3.29^39^.

## Results

### Behaviour

Because shock outcomes were predetermined, the reinforcement learning task as presented to participants was intrinsically unlearnable. Nevertheless, evidence that participants engaged in reinforcement learning could be assessed by comparing the frequency with which participants took actions congruent with the prior outcome (win-stay, lose-shift) compared to actions that were incongruent (win-shift, lose-stay). There was strong evidence of such outcome sensitivity across the 200 trials: ${\overline{X}}_{D}$ = 20.73, ${s}_{D}$ = 23.04, 95% CI [13.72, 27.73], paired *t* = 5.97, df = 43, *p* < 0.001, *d* = 0.90) Additionally, when queried at each block end as to which fractal in a pair was associated with the smaller amount of shock (ten queries over the experiment, corresponding to five blocks × two stakes), participants were successful for more pairs than not: ${\overline{X}}_{D}$= 2.59, ${s}_{D}$ = 3.07, 95% CI [1.66, 3.52], paired *t* = 5.60, df = 43 *p* < 0.001, *d* = 0.84).

### RPE encoding

**Figure 2:**
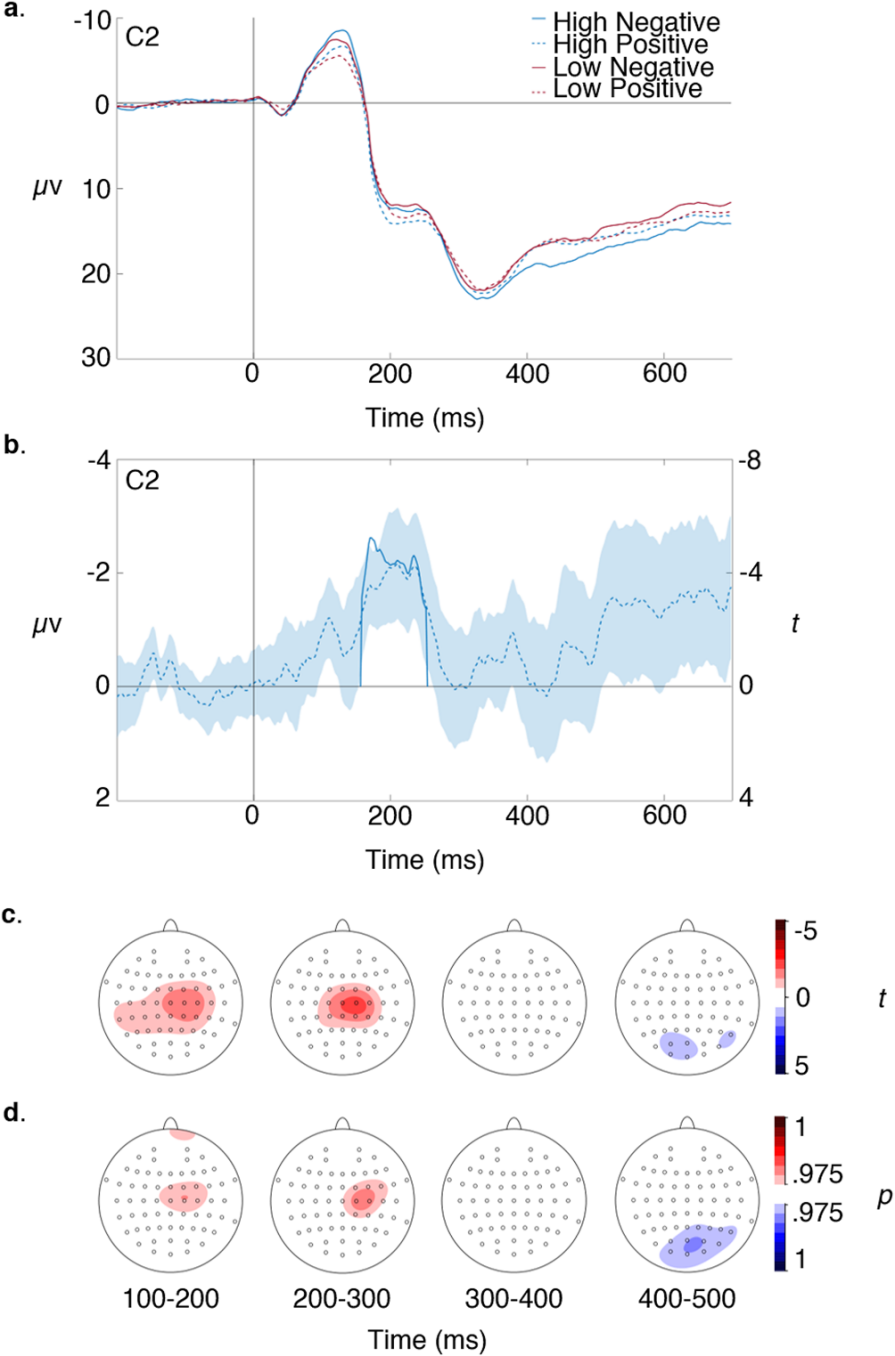
(**a**) Simple waveforms. (**b**) FRN (i.e. LN–HP difference wave) expressed as voltage (dotted line) and thresholded *t* statistic over participants (bold line), with shading showing two standard errors of the mean. (**c**) Scalp topography of the thresholded FRN *t* statistic showing areas where increased RPE value is encoded with positive voltage (red) and negative voltage (blue). (**d**) Scalp topography of thresholded Bayesian contrasts showing same effects. All plots were created in MATLAB version 2020a (https://uk.mathworks.com/products/matlab.html).

Simple grand average waveforms for the four conditions, and the LN–HP difference wave are shown in Fig. 2a, b, both plotted at C2 where the difference wave was maximal. Figure 2c shows the scalp topography of this difference wave at points where it was significant after cluster randomisation and reveals two significant clusters for the effect. The first occurs in the interval 150–260 ms, maximal at C2 at 210 ms (Monte Carlo *p* = 0.000035), with positive RPEs associated with positive voltage, thus corresponding with the conventional FRN. The second, weaker effect, occurs in the interval 390–480 ms, maximal at POz at 418 ms (Monte Carlo *p* = 0.00053), with positive RPEs associated with negative voltage. Figure 2d shows the topography of RPE encoding under Bayesian *t* tests, thresholded at 0.975. This reveals a very similar pattern, with an effect in the interval 150–250 ms, maximal at C2, with positive RPEs associated with positive voltage, and an effect in the interval 390–480, maximal at P8, with positive RPEs associated with negative voltage. Supplementary Fig. S2 presents analogous scalp plots for the effect of physical salience.

### Startle response

Startle responses occurred with similar frequency in high stakes ($\overline{X}$ = 9.14, *s* = 5.25) and low stakes conditions ($\overline{X}$ = 9.00, *s* = 4.65). The startle response was maximal 120 ms after noise onset. Supplementary Fig. S3 shows the time course of the grand average startle response separately for high and low stakes conditions, weighted by the number of startle response trials shown by each participant. The startle response was of significantly greater positive amplitude for high stakes compared to low: ${\overline{X}}_{D}$ = 0.91 μV, ${s}_{D}$ = 2.89, 95% CI [0.01, 1.81], paired *t* = 2.04, df = 41, *p* = 0.048, *d* = 0.31. A correlation of startle response amplitude at 120 ms (collapsing out stakes conditions) and average FRN amplitude, as measured in the interval 150–260 ms at C2, showed greater startle response to be significantly associated with greater FRN amplitude (Pearson’s *r* = − 0.37, *p* = 0.013, Spearman’s rho = − 0.32, *p* = 0.028, N = 44). Very similar values were found when the 120 ms point sample of startle response amplitude was replaced with mean startle response activity in an interval 100–140 ms following noise onset.

## Discussion

The present study addressed a dearth of research on event related potentials generated by RPEs associated with unconditioned stimuli. It provided evidence for an RPE-encoding component that responded to an unconditioned stimulus with the same polarity and topography as the conventional cue-locked FRN, and which occupied the earlier portion of that component’s typical interval. It thus provides provisional evidence that the FRN encodes the value of primary reinforcers and is not elicited simply by arbitrary “truth cues”.

Timing differences between the component elicited here and the typical cue-locked FRN were apparent. The component’s onset of 150 ms corresponded to that shown by the cue-locked FRN, but it peaked and ended some 70 ms earlier than is typically observed. This is not expected from temporal difference learning, which predicts no difference in the neural response to RPEs carried by unconditioned stimuli and the conditioned stimuli that cues should develop into over the course of learning. The truncated nature of the component raises the possibility that it shares some, but not all of the neural activity underlying the cue-locked FRN. The principal task difference in the current study was that at no point in the experiment were participants required to maintain in working memory an association between a cue and a primary reinforcer. This contrasts with cue-based experiments. While the cue-reinforcer mapping may be discarded in time as associative learning confers value directly onto the cue (i.e. making it a conditioned stimulus), waveforms averaged over the experiment will incorporate a number of trials where this mapping was in effect.

One possibility is that in cue-based experiments the RPE is delayed while associative mappings held in working memory are passed to the midbrain DA neurons responsible for RPE generation. The fact that the component observed here is topographically similar to the typical cue-locked FRN is consistent with a common process. This is supported by an fMRI study showing that when truth cues indicating success change every trial, preventing any possibility of their acquiring value via associative learning, the midbrain still appears to activate, presumably from cortical input carrying these mappings^40^. Such an explanation speaks to the interesting possibility that all RPE computation is obligatorily dependent on a single anatomically defined pathway into which diverse inputs feed. The actual point of confluence of these inputs would remain an open question. While this might be the midbrain, mappings might instead converge in the striatum, which has been proposed to serve a generalised gating role for prediction errors^41^. This question would be difficult to resolve by the event related potential method which cannot detect midbrain activity and is limited in its access to subcortical structures such as the striatum.

The account above suggests that the delayed peak in the typical cue-locked FRN, relative to that shown in the present experiment, reflects the interposition of a preliminary step prior to RPE encoding. An alternative explanation is that the delayed cue-locked FRN reflects the *addition* of later, functionally separate processes that happens to show the same polarity at frontocentral sites and whose spatial characteristics are unresolvable by the coarse spatial resolution of EEG. The midbrain RPE signal is typically regarded as an example of model-free reinforcement learning, but cue-locked FRN experiments entail a degree of instruction-based learning, in the form of truth cues, which can be regarded as a prime example of goal-directed, or model-based learning^42^. Both such signals may contribute to the cue-locked FRN, with one study using principal components analysis to decompose the FRN into separate model-based and model-free RPEs^43^.

Some limitations should be noted. In the present study, RPEs were studied only in the aversive domain, owing to the difficulty of finding an appetitive unconditioned stimulus that could be delivered with temporal precision. It is thus possible that the shortened latency is specific to the aversive domain. An influential single cell study^9^ found that, while neurons responding to juice delivery were the same as those responding to the conditioned stimuli predicting juice delivery, separate cell populations responded for air puff delivery compared to the conditioned stimuli predicting air puff. Furthermore, the negative RPE signal carried by phasic reduction in cell firing occurred much earlier in response to air puff compared to conditioned stimuli for air puff (51 ms vs. 179 ms).

A further consequence of restricting the design to the aversive domain is that it cannot formally distinguish between coding of RPE and motivational salience. The motivational salience of a reinforcer lies in its ability to elicit attention due to its motivational relevance without regard to its actual valence^20^. Appetitive and aversive events thus produce the same response in a motivational salience encoder to the extent that these events are equally motivationally relevant. The LN–HP difference wave, which controls out effects of physical salience, has been interpreted in this study as indicating RPE encoding. However, the LN outcome constitutes greater motivational salience than the HP outcome since, while both outcomes are equally unexpected (*p* = 0.5), the LN outcome constitutes expectancy violation via an excess rather than deficit with respect to the expected amount of a motivational good (shock). The significant LN–HP difference wave observed might thus indicate a component coding for motivational salience via voltage negativity rather than an RPE via voltage positivity. Distinguishing between the two interpretations requires a design that varies the size of prediction errors for both appetitive and aversive goods. If the negative voltage shift we have observed as an aversive stimulus becomes more intense was also found when an appetitive stimulus becomes more intense, our findings would be interpreted as showing motivational salience encoding. If increasing appetitive stimulus intensity produced a positive shift then the RPE explanation would be retained. In fact, cue-locked experiments that have included this manipulation have provided good evidence that motivational salience *is* encoded in the earlier portion of the interval associated with the FRN^44–47^. However, this encoding is carried by a voltage positivity and for this reason we can be confident that this is not the component elicited here. It is thus more parsimonious to assume the observed component is the same, or related, RPE encoding seen in other FRN studies than a new salience component of opposite polarity to that usually seen. Nevertheless, it remains the case that this possibility cannot be ruled out.

Within the aversive domain, the present experiment uses a single aversive reinforcer, shock. Because the number of experiments using shock to generate prediction error is so few, it is not yet clear whether shock has specific effects on the waveform, once its sensory effects are controlled out to isolate the underlying prediction error. Three cue-locked shock studies all reported later FRNs than that shown here, but were themselves highly variable (250 ms^45^, 380 ms^48^, 420 ms^49^). Task effects are likely to have contributed to this variability, in particular whether participants were active or passive.

Using startle response in the context of a conditioned Pavlovian stimulus (the stakes cue), this study found modest evidence of a relationship between an affective physiological response and the expected strength of a forthcoming shock. This accords with other studies showing correlations between expected value (here, negative value) and physiological measures such as startle response^30^ and pupil dilation^50,51^. Additionally we found that, measured across participants, the average strength of the startle response incurred to noise bursts was correlated with the average amplitude of the FRN produced by instrumentally unrelated electric shocks. This effect was modest, especially when taken in the context of the low power present in between-subjects comparisons and should be viewed with caution. However, it provides provisional evidence, albeit circumstantially, for an affective element in RPE generation. Such a relationship is of course implied in the phenomenon of Pavlovian-instrumental transfer, in which the vigour of simple instrumental responses is increased and decreased by appetitive and aversive Pavlovian cues unrelated to the stimulus–response relationship. In humans, fMRI studies have suggested the striatum to be an important site of Pavlovian-instrumental transfer^52,53^. An intact nucleus accumbens is necessary for startle attenuation^54^, and this structure is also a major projection site for DA neurons implicated in RPE transmission. It appears to serve the critic role in an actor-critic architecture shared with the dorsal striatum and is regarded as a “limbic–motor interface^55–57^, allowing Pavlovian incentive value to modulate instrumental behaviour. RPEs serve learning rather than behaviour however, raising the question of why affect might modulate them. One possibility is that this indicates an architecture that biases learning based on the profitability of the environment^58^, with this profitability carrying an affective character. Indeed, FRN amplitudes appear to be reduced for high anxiety people^59,60^ suggesting a reluctance to positively adjust expectations in the face of transient positive outcomes, or a seizing of negative outcomes as evidence of poor chances of future success.

In conclusion, the present study elicited a component resembling the FRN in response to an unconditioned stimulus. The finding that an FRN is generated in response to unconditioned as well as conditioned stimuli strengthens the claim that it reflects the transmission of an RPE from midbrain DA cells. However, the relatively short latency for the FRN to the unconditioned stimulus used here suggests that the typically observed cue-locked FRN may incorporate neural processes other than those carried by midbrain DA neurons.

## Supplementary Information

Supplementary Information.

## Data Availability

All supporting data is available on the Open Science Framework at the following link: https://osf.io/9xc3r/?view_only=3eeb440fcc1b4762ab3be9416d25af22.
