# Supplementary material for: Reward prediction error in the ERP following unconditioned aversive stimuli
Source: Sci Rep. 2021 Oct 7;11:19912. doi: 10.1038/s41598-021-99408-4 (PMC8497484; doi:10.1038/s41598-021-99408-4)
Supplement: Supplementary file 1 — Supplementary Information. [file 41598_2021_99408_MOESM1_ESM.pdf]

Reward prediction error in the ERP following unconditioned aversive stimuli

Harry J. Stewardson*
Thomas D. Sambrook

*Corresponding author
School of Psychology, University of East Anglia,
Norwich Business Park, NR4 7TJ, United Kingdom
Tel +44 1603 597753
Fax +44 1603 597756

Supplementary Information

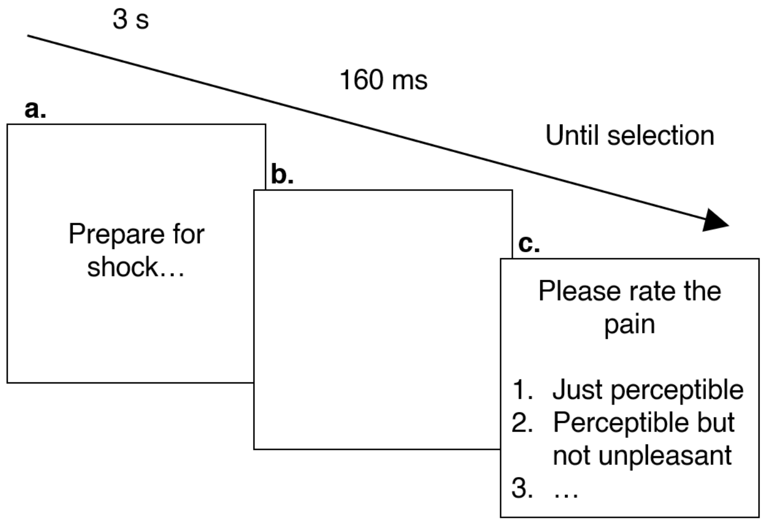

Supplementary Figure 1. One trial of the shock calibration procedure. a. Warning for the approaching shock. b. Shock is delivered. c. Participant rates the pain: “1” (“just perceptible”), “2” (“perceptible but not unpleasant”), “3” (“very slightly unpleasant”), “4” (“slightly unpleasant”), “5” (“moderately unpleasant”), “6” (“unpleasant”), “7” (“highly unpleasant but still tolerable for the forthcoming experiment”), “8” (“intolerable for the forthcoming experiment”), “9” (“very painful indeed and intolerable for the forthcoming experiment”), or “10” (“excruciating and intolerable for the forthcoming experiment”).

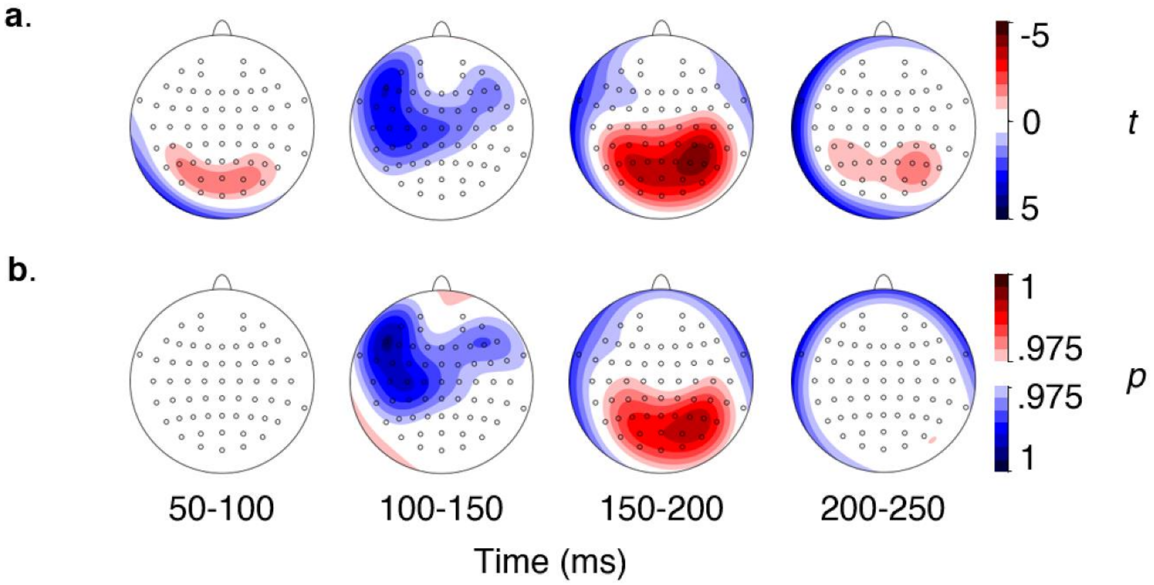

Supplementary Figure 2 a. Scalp topography of physical salience encoding expressed as the thresholded t-statistic for the difference wave (LP + LN) – (HP + HN): blue indicates encoding of increasing physical salience via voltage negativity, red indicates encoding via voltage positivity. b. Scalp topography of thresholded Bayesian contrasts showing physical salience encoding via the same colour mapping.

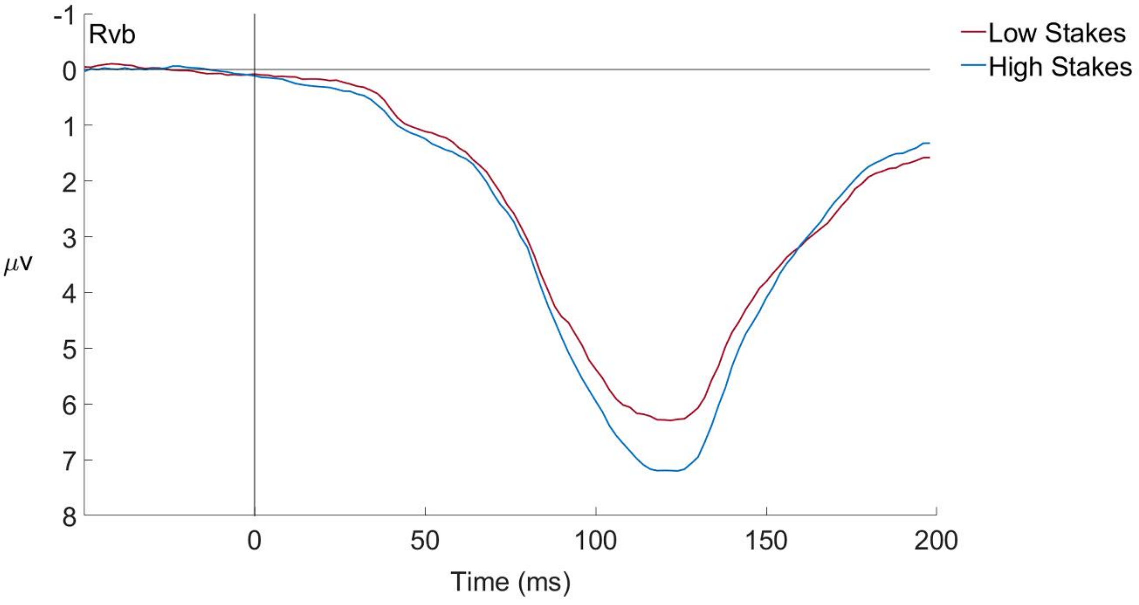

Supplementary Figure 3. Grand average startle response weighted by the number of startle response trials shown by each participant
